# Oxygen and Cell Fate Decisions

**DOI:** 10.4137/grsb.s434

**Published:** 2008-02-10

**Authors:** Qun Lin, Yuri Kim, Rodolfo M. Alarcon, Zhong Yun

**Affiliations:** 1 Department of Therapeutic Radiology, Yale University School of Medicine, New Haven, CT 06520, U.S.A; 2 Air Force Research Laboratory, Brooks City-Base, Texas 78235-5107, U.S.A

**Keywords:** adipogenesis, chondrogenesis, differentiation, hypoxia, myogenesis, oxygen, placenta, preadipocytes, progenitor cells, stem cells, trophoblasts

## Abstract

Molecular oxygen has been known to play a critical role in a wide range of biological processes including glycolysis, mitochondrial respiration, angiogenesis, pulmonary functions, and cardiovascular activities. An emerging theme has developed in recent years that oxygen has significant impact on embryonic development, maintenance of stem cells, and cellular differentiation or cell fate decisions. Among the notable observations, early embryonic development takes place in a hypoxic microenvironment. Hematopoietic stem cells appear to be located in hypoxic regions within the bone marrow. Majority of the current observations have shown that hypoxia seems to prevent cellular differentiation and to maintain pluripotency of stem/progenitor cells. Genetic studies have demonstrated a critical role of hypoxia-inducible factors 1α and 2α in embryonic development. These intriguing observations demonstrate an important role of molecular oxygen in such fundamental biological processes as stem cell maintenance and regulation of cell fate decisions. Herein, we describe some of the latest advances in the biology of molecular oxygen and provide our perspectives on the potential impact of these interesting findings.

## Introduction

Molecular oxygen (O_2_) is an essential element for life on earth. It is the ultimate electron acceptor in the mitochondrial electron transport chain and is the only cellular nutrient in a gaseous form. A large family of oxidoreductases, such as prolyl hydroxylases and cytochrome c-oxidase use O_2_ as a substrate (Ozer and Bruick, 2007; Vanderkooi et al. 1991). Increasing amounts of evidence also indicate that O_2_ functions as a signaling molecule, regulating a wide range of biological processes including erythropoiesis, angiogenesis, energy metabolism, and cellular differentiation. Over the past 5–10 years, a new role of oxygen has emerged as an important signaling molecule for the regulation of stem cell maintenance and cell fate decisions.

In the current geological era, the atmospheric O_2_ level reaches approximately 21% at sea level, but reduces with increasing elevation. Living organisms, especially mammals, rely on sophisticated respiratory-circulatory systems to constantly deliver O_2_ to tissues in order to sustain cellular functions and viability. Hypoxia occurs when tissue oxygenation decreases to a certain level, which can result from pulmonary obstruction, cardiovascular malfunction, severance of blood vessels, and growth of solid tumors. Nonetheless, hypoxia is only an operational term because physiological levels of O_2_ partial pressures (pO_2_) vary from tissue to tissue (Vaupel et al. 2007). Based on consensus in current literature, hypoxia is considered as pO_2_ ≤ 2% at which the widely used hypoxia marker, the hypoxia-inducible factor-1α (HIF-1α) is robustly stabilized and becomes competent for activation transcription of target genes.

The hypoxia-inducible factor-1 (HIF-1) pathway is the best-studied molecular mechanism of O_2_ homeostasis in higher eukaryotes. HIF-1 is a heterodimeric transcription factor consisting of HIF-1α and HIF-1β, both of which are members of the basic helix-loop-helix *Per*, *AhR* and *Sim* (bHLH-PAS) family (Semenza, 2000; Wang et al. 1995). Under physiological normoxia, HIF-1α protein becomes hydroxylated at two proline residues located in its O_2_-dependent degradation domain (Ivan et al. 2001; Jaakkola et al. 2001) and is targeted by the von Hippel-Lindau (VHL) protein for ubiquitination and proteosome-mediated degradation (Maxwell et al. 1999; Ohh et al. 2000). Under hypoxia, HIF-1α protein is not hydroxylated. The stabilized HIF-1α translocates into the nucleus where it dimerizes with the O_2_-independent HIF-1β and initiates gene transcription by binding to hypoxia-responsive enhancer elements (HRE) with the consensus sequence of 5′-ACGTG-3′ (Harris, 2002; Semenza, 2000). Genes induced by HIF-1 are involved in a wide range of cellular functions such as cell growth, survival, motility, angiogenesis, energy metabolism, and cellular differentiation (Harris, 2002; Lin et al. 2006; Semenza, 2000; Yun et al. 2005; Yun et al. 2002). As a homologue of HIF-1α, the endothelial PAS domain protein (EPAS), now known as HIF-2α, also interacts with HIF-1β. HIF-2α shares the same mechanism of O_2_ regulation with HIF-1α but seems to have limited tissue distribution (Ema et al. 1997; Tian et al. 1998; Wiesener et al. 2003). Further discussion of the HIF pathway can be found in many excellent reviews that have dealt with this subject in great detail. This review will provide a brief account on our current understanding of the role of O_2_ in the regulation of cellular differentiation.

## Hypoxia and Embryonic Development

The embryonic development at early stages likely occurs in a physiologically hypoxic microenvironment. During the first trimester, the average pO_2_ level in human placentas is approximately 18 mmHg (2.4%) as measured by a polarographic O_2_ microelectrode, which is significantly lower than average pO_2_ of 40 mmHg or 5.3% in adjacent endometrium (Rodesch et al. 1992). An independent study has also shown that the placental pO_2_ is 2.5 fold lower than the decidual pO_2_ before the 11th week of gestation in humans (Jauniaux et al. 2001). It is therefore conceivable that pO_2_ in the developing embryo would be lower than 2%. The importance of cellular response to hypoxia in embryonic development and differentiation has been clearly demonstrated in genetic mouse models. Homozygous deletion of either *HIF-1α* or *HIF-1β* is found to be embryonically lethal in mice. The *HIF-1α*^−/−^ mouse embryos succumb during midgestation around 10 days post coitus (d.p.c) to loss of mesenchymal cells and impaired cardiovascular development (Carmeliet et al. 1998; Iyer et al. 1998; Ryan et al. 1998). The *HIF-1β*^−/−^ mouse embryos die by 10.5 d.p.c due to vascular deficiencies in the yolk sac and/or placenta (Kozak et al. 1997; Maltepe et al. 1997). However, mouse phenotypes caused by homozygous deletion of *HIF-2*α vary depending on the strains used for generation of *HIF-2*α ^−/−^ mice. Prominent phenotypes include embryonic lethality due to cardiovascular defects, embryonic and postnatal death due to mitochondrial abnormalities, and perinatal lethality resulting from impaired pulmonary development (Compernolle et al. 2002; Peng et al. 2000; Scortegagna et al. 2003; Tian et al. 1998). Nonetheless, it has been shown genetically that HIF-2α directly enhances the transcription of *Oct-4* during embryonic development by binding to the *Oct-4* gene promoter (Covello et al. 2006). These genetic models suggest that *HIF-1α* is essential for early embryonic development, whereas *HIF-2α* may be required for late fetal development of certain tissue types.

The establishment of uteroplacental circulation relies on cytotrophoblast invasion into the uterine spiral arterioles. A large body of evidence has shown that proliferation, invasion and differentiation of cytotrophoblasts are tightly regulated along the pO_2_ gradient from the anchoring villi in the inner placental space with low pO_2_ to the uterine spiral arterioles with high pO_2_ (Caniggia et al. 2000; Genbacev et al. 1997; James et al. 2006). In the low pO_2_ microenvironment, cytotrophoblasts proliferate as extravillous trophoblasts with a poorly differentiated phenotype and invade into the surrounding tissue. As they enter into the proximity of O_2_-rich uterine spiral arterioles, the proliferating extravillous trophoblasts differentiate into a highly invasive phenotype and penetrate into the arterial endothelium.

However, it remains to be clearly understood what role the HIF pathway plays in the regulation of growth and differentiation of trophoblasts. Caniggia et al. analyzed HIF-1α expression in human placenta sections at different gestational stages using *in situ* hybridization and RT-PCR (Caniggia et al. 2000). Levels of *HIF-1α* mRNA are high between 5–8 weeks of gestation, but decline in the 9th week of gestation. Expression of *HIF-1α* mRNA is robust in the trophoblast layers but much weaker in the mesenchyme (Caniggia et al. 2000). By Northern blotting analysis, Rajakumar and Conrad have found that *HIF-1α* mRNA does not change significantly with the gestational stage, whereas *HIF-2α* mRNA markedly increases with advancing gestation (Rajakumar and Conrad, 2000). Nonetheless, both HIF-1α and HIF-2α proteins decrease with gestational age (Rajakumar and Conrad, 2000). The overall dynamic change of HIF-1α and HIF-2α proteins coincides with increased oxygenation at the end of the first trimester.

Surprisingly, Maltepe et al. have recently shown that both *HIF-1α* and *HIF-2α* mRNA increase dramatically during *in vitro* differentiation of trophoblast stem cells, and the increased transcription of *HIF-1α* and *HIF-2α* mRNA occurs at 21% O_2_ (Maltepe et al. 2005). Increasing amounts of HIF-1α and especially HIF-2α proteins are also found in differentiated trophoblast cells (Maltepe et al. 2005). The observations by Maltepe et al. certainly do not agree with those by Caniggia et al. and by Rajakumar and Conrad as discussed above. The discrepancies are likely to be caused by different experimental approaches. While others used *ex vivo* human placental tissues, Maltepe et al. used an *in vitro* model of trophoblast stem cells initially derived from mouse embryonic fibroblasts. Therefore, the contradictory observations could potentially be attributed to differences between human and mouse tissues and/or among different cellular origins.

Nevertheless, the consensus of these studies is that HIF-2α seems to be regulated by a different mechanism in trophoblasts in contrast to HIF-1α. Expression of *HIF-2α* mRNA (Maltepe et al. 2005; Rajakumar and Conrad, 2000) and HIF-2α protein (Maltepe et al. 2005; Rajakumar and Conrad, 2000) increases in trophoblasts with gestational age, while *HIF-1α* mRNA remains largely unchanged. Consistent with these observations, expression of *HIF-2α* mRNA increases with adipogenic differentiation and HIF-2α protein is regulated by O_2_-independent mechanisms in adipocytes (Lin et al. 2006). Such differential regulation of HIF-1α and HIF-2α may reflect their pleiotropic functions during cellular differentiation. Genetic models will be required to delineate the functions of *HIF-1α* and *HIF-2α* during placental development and trophoblast differentiation.

The role of HIF-1 in lymphocyte development has been studied using an *RAG-2*^−/−^ blastocyst complementation assay (Kojima et al. 2002; Kojima et al. 2003). In chimeric mice generated by injecting *HIF-1α*^−/−^ embryonic stem cells into *RAG-2*^−/−^ blastocysts, the BM-derived *HIF-1α*^−/−^ B-cells were developmentally blocked in the late pre-B stage (Kojima et al. 2002). In contrast, T-cell development in the thymus of *HIF-1α*^−/−^*/RAG-2*^−/−^ chimeric mice is not affected, nor is the extra-medullar B cell development (Kojima et al. 2002; Kojima et al. 2003). These observations suggest that HIF-1α plays a differential role in development of B and T cells.

## Hypoxia and Differentiation of Bone Marrow-Derived Stem Cells

Early studies of hematopoietic regeneration after bone marrow (BM) ablation by ionizing radiation revealed the existence of a radioresistant population of hematopoietic stem cells (HSC) (Maloney and Patt, 1968; Rubin et al. 1977). The radioresistance of these HSCs may reflect their existence in a relatively hypoxic microenvironment with lower pO_2_ than other well-vascularized and well-oxygenated areas (Allalunis et al. 1983). In healthy human volunteers, the mean hemoglobin O_2_ saturation in BM aspirates was found to be about 87.5%, much lower than the 99% hemoglobin O_2_ saturation in peripheral blood (Harrison et al. 2002). Using an oxygen microelectrode to directly measure tissue pO_2_ *in situ*, Ceradini et al. have found that the mean pO_2_ in mouse BM is around 18 mmHg or 2.4% O_2_, as compared to about 34 mmHg (4.5% O_2_) in adjacent non-ischemic muscle (Ceradini et al. 2004). A recent study has shown that the highest concentration of HSCs is contained in the cell population with the lowest perfusion, as shown by the lowest fluorescence of the vessel-permeating dye Hoechst 33342 (Parmar et al. 2007), suggesting that HSCs are likely localized in regions distant from blood vessels. The hypoxic nature of HSCs is further demonstrated by their selective binding of the hypoxia-activated compound pimonidazole and their increased sensitivity to the hypoxia-activated cytotoxin tirapazamine (Parmar et al. 2007). These data strongly support the concept that HSCs are localized in hypoxic regions of bone marrow. Nonetheless, the exact location of such hypoxic HSC niches needs to be clearly determined by immunohistological analysis at cellular levels *in vivo*.

The multipotency of HSCs seems to be maintained by hypoxia. Using non-adherent murine BM-derived cells, Cipolleschi et al. found that the marrow-repopulating potential of HSCs cultured at 1% O_2_ even without stromal cells was higher than that of HSCs cultured under ambient tissue culture conditions (Cipolleschi et al. 1993), suggesting that the hypoxic culture contains more pluripotent stem cells than the normoxic culture. Because HSCs lose their pluripotency quickly when maintained under normal tissue conditions, it is also possible that hypoxia maintains pluripotency of HSCs. Consistent with these findings, Ivanovic et al. showed that hypoxia maintained both the colony-forming and marrow-repopulating potential of murine BM cells better than normoxia did (Ivanovic et al. 2000; Ivanovic et al. 2002). Danet et al. investigated the effect of hypoxia (1.5% O_2_) on the ability of human lin^−^CD34^+^CD38^−^ HSCs to reconstitute the BM of lethally irradiated severe-combined immunodeficient (*SCID*) mice (Danet et al. 2003). Using a serial dilution assay, they showed that *SCID*-repopulating cells were increased 5.8 fold in hypoxic cultures, as compared to cells grown at normoxia (Danet et al. 2003). A recent proteomics study has further shown that LSK (lin^−^ Sca-1^+^ c-Kit^+^) HSCs preferentially displayed a proteomic profile reminiscent of hypoxia-regulated gene expression (Unwin et al. 2006). These observations strongly suggest an important role for hypoxia in the maintenance of HSC pluripotency.

HSCs are located in a special microenvironment, called the stem cell niche, where their stem cell phenotype and differentiation are tightly regulated via interactions with the supporting stromal cells (Nagasawa, 2006; Wilson and Trumpp, 2006; Yin and Li, 2006). As discussed above, these HSC niches are likely to be hypoxic. Therefore, niche stromal cells are also under the influence of hypoxia. Marrow stromal cells can be grown *in vitro* as pluripotent mesenchymal stem cells capable of differentiating into osteoblasts, adipocytes and chondrocytes. D’Ippolito et al. have shown that hypoxia (1%–3% O_2_) inhibits the differentiation and enhances pluripotency of a human BM stromal cell line (D’Ippolito et al. 2006). When exposed to hypoxia, human BM-derived stromal cells increase expression of a subset of genes normally found in embryonic cells such as *OCT-4* and *Rex-1* by RT-PCR (D’Ippolito et al. 2006; Grayson et al. 2006), as well as cell-surface marker SSEA-4 by fluorescence-assisted cell sorting or FACS (D’Ippolito et al. 2006). Even short-term exposure to hypoxia seems to enhance pluripotency of BM-derived stem cells. Martin-Rendon et al. and Grayson et al. have shown that hypoxia-preconditioned human BM-derived stem cells exhibit higher colony forming units and increased differentiation potential towards chon-drogenic, adipogenic or osteogenic differentiation (Grayson et al. 2007; Grayson et al. 2006; Martin-Rendon et al. 2007).

Current evidence suggests that HIF-1 may be directly involved in the regulation of BM-derived stem and progenitor cells. Both HSCs and BM stromal cells express HIF-1α (D’Ippolito et al. 2004; Danet et al. 2003; Okuyama et al. 2006). In BM stromal cells, HIF-1 is directly involved in the enhanced expression of *VEGFR1* by hypoxia (Okuyama et al. 2006). The expression of stromal-derived factor-1 (*SDF-1*) is upregulated by HIF-1 and plays an important role in hypoxia-induced trafficking of BM-derived progenitor cells (Ceradini et al. 2004). However, mechanisms of hypoxia-signaling in BM-derived stem cells are likely to complex involving both HIF-dependent and -independent pathways that remain to be fully understood.

Maintaining BM stromal cells in a stem cell-like state may have significant ramifications for maintenance of HSCs. Studies using transgenic mice have shown that ablation of osteoblasts at an early stage of osteoblastogenesis results in a severe decrease in BM HSCs (Visnjic et al. 2004), whereas loss of osteoblasts at later stages of differentiation has no effect on hematopoiesis (Corral et al. 1998). These data suggest that immature stromal cells are better suited for the maintenance of HSCs (Wilson and Trumpp, 2006). It is thus conceivable that hypoxia contributes to the establishment of an undifferentiated niche microenvironment that prevents inopportune differentiation of HSCs.

## Hypoxia and Differentiation of Mesenchymal Stem/Progenitor Cells

### Adipogenic differentiation

Several *in vitro* studies have shown that adipogenic progenitor cells are prevented from undergoing differentiation by hypoxia (Kim et al. 2005; Lin et al. 2006; Sahai et al. 1994; Yun et al. 2002). It is worth mentioning that inhibition of adipogenic differentiation occurs even at 1%–2% O_2_, a physiologically relevant level of hypoxia (Lin et al. 2006; Yun et al. 2002). Interestingly, these progenitor cells remain undifferentiated and uncommitted under hypoxic conditions and can still undergo adipogenic differentiation once they return to normoxic conditions (Lin et al. 2006). These data suggest that hypoxia has the ability to maintain stem cell functionality by arresting stem/progenitor cells in an undifferentiated state. This finding provides a reasonable explanation as to why a hypoxic niche may potentially be critical for the maintenance of stem cells *in vivo*.

Mechanistically, hypoxia represses the expression of *PPARγ2* and *C/EBPα,* two critical differentiation-determination genes during adipogenic differentiation (Lin et al. 2006; Yun et al. 2002). Therefore, hypoxia prevents progenitor cells from committing to terminal adipogenic differentiation. HIF-1α is expressed in both progenitor cells and differentiated adipocytes, whereas HIF-2α is only detected in mature adipocytes (Lin et al. 2006; Shimba et al. 2004). These data suggest that HIF-1α is more involved in the regulation of adipogenic progenitor cells whereas HIF-2α may be more important in mature adipocytes. Indeed, HIF-1α plays an essential role in inhibiting adipogenic differentiation. When *HIF-1α* is knocked down by gene-specific siRNA, progenitor cells become capable of adipogenic differentiation under hypoxic conditions. On the other hand, ectopic expression of constitutively active *HIF-1α* mutants results in inhibition of adipogenic differentiation under normoxic conditions (Lin et al. 2006).

Further downstream of the HIF-signaling pathway, the hypoxia-induced gene *DEC1/Stra13* is directly involved in inhibition of *PPARγ2* transcription. *DEC1/Stra13*, also referred to as *BHLHB2*, *SHARP2* and *Clast5*, is a putative transcription repressor and contains an N-terminal basic helix-loop-helix (bHLH) domain homologous to those of the *Hairy* and *Enhancer-of-Split* (HES) family. Studies have shown that HIF-1 is required for hypoxic induction of *DEC1/Stra13* transcription (Miyazaki et al. 2002; Wykoff et al. 2000; Yun et al. 2002). Ectopic expression of *DEC1/Stra13* results in decreased *PPARγ2* transcription and inhibition of adipogenic differentiation (Yun et al. 2002). These data demonstrate that signal transduction mediated by HIF-1 plays a critical role in the regulation of adipogenic differentiation.

Other hypoxia-regulated signaling pathways may also contribute to the inhibition of adipogenic differentiation. In human BM-derived stromal cells, hypoxia can activate the Transforming Growth Factor β (TGFβ)-Smad pathway by increasing levels of phosphorylated Smad2/3, which results in inhibition of adipogenic differentiation (Zhou et al. 2005). It will be interesting to see whether the canonical HIF-pathway is involved in activation of the TGFβ-Smad pathway. We have also found that hypoxia increases the expression of the stem/progenitor marker pref-1/DLK1 (Lin et al. 2006), a negative regulator of adipogenic differentiation (Smas and Sul, 1993; Wang et al. 2006). Enhanced expression of pref-1/DLK1 in adipogenic precursor cells is independent of HIF-1 (Lin et al. 2006). These observations demonstrate that both HIF-dependent and HIF-independent pathways are involved in repression of adipogenic differentiation.

### Myogenic differentiation

Hypoxia also inhibits the differentiation of myogenic progenitor cells (Gustafsson et al. 2005; Yun et al. 2005). However, inhibition of myofiber formation depends on the degree of hypoxia, with strongest inhibition at nearly anoxic pO_2_ level (Yun et al. 2005). Hypoxia inhibits expression of the key myogenic transcription factor *MyoD* and, to a lesser degree, the transcription coactivator *E2A* (Yun et al. 2005). Interestingly, MyoD expression is only transiently blocked at 0.5%–2% O_2_, but gradually recovers even when cells are kept under hypoxic conditions. Consequently, myogenic differentiation manages to adapt to persistent or chronic hypoxia (Yun et al. 2005).

In contrast to its role in adipogenesis, HIF-1α does not seem to play a significant role in the regulation of myogenic differentiation. Ectopic expression of constitutively active HIF1α does not change myogenesis under normoxia or hypoxia (Yun et al. 2005). Contrary to the above results, Gustafsson et al. have reported that HIF-1α is involved in inhibition of myogenesis by interacting with the Notch intracellular domain (NICD) and subsequently activating downstream genes of Notch signaling (Gustafsson et al. 2005). However, Yun et al. have found that expression of *Notch1*, *Notch2* and *Notch3* is not significantly affected at 0.5% O_2_, but is markedly reduced at 30.01% O_2_ (Yun et al. 2005). Consistently, the level of endogenous NICD protein is not significantly affected at 1%–2% O_2_, but is again dramatically reduced at <0.01% O_2_. Furthermore, treatment of C2C12 myoblasts with N-[N-(3,5-difluorophenylacetyl-L-alanyl)]-S-phenylglycine t-butylester (DAPT), a specific γ-secretase inhibitor, to block Notch signaling by preventing NICD formation has no significant effect on myogenic differentiation under normoxic or hypoxic conditions (Yun et al. 2005). The observation by Yun et al. suggests that Notch signaling is not likely to be involved in the inhibition of myogenic differentiation. Although both studies use the C2C12 model, the inconsistent observations might potentially be attributed to differences in maintenance of tissue culture and experimental conditions such as differentiation and hypoxia. The exact mechanisms by which hypoxia regulates myogenic differentiation remain to be investigated.

### Chondrogenic differentiation

Cartilage tissue is avascular and contains regions of hypoxia under normal physiological conditions (Brighton and Heppenstall, 1971a, b). During embryonic development, precartilaginous condensation of mesenchymal cells occurs in an avascular microenvironment with gross hypoxia as revealed by strong staining of hypoxia-activated nitroimidazole compounds, and by immunochemical staining of HIF-1α (Provot et al. 2007; Robins et al. 2005). Condensed mesenchymal stem cells thus differentiate into chondrocytes for joint formation. Genetic mouse model studies using conditional knockout of *HIF-1α* have clearly demonstrated a critical role of *HIF-1α* in chondrogenic development. When *HIF-1α* is deleted in late stage chon-drocytes using the *Col2α1* promoter-drive *Cre* approach, survival of chondrocytes is severely reduced (Schipani et al. 2001). When *HIF-1α* is deleted in early stage of chondrogenesis using the *Prx1* promoter-drive *Cre* approach, embryos develop abnormal cartilaginous primodia and impaired joint formation (Amarilio et al. 2007; Provot et al. 2007). Expression of *Sox9*, a key regulator of chondrogenesis, is reduced in *HIF1α-*deleted limbs (Amarilio et al. 2007). HIF-1α regulates *Sox9* transcription by directly binding to *Sox9* promoter (Amarilio et al. 2007). These studies have provided strong evidence that the HIF-1 pathway is critically involved in chondrogenic differentiation during embryonic development. These observations also suggest that mesenchymal stem cells may still be able to commit to chondrogenic differentiation under hypoxic conditions *in vivo*.

Surprisingly, Malladi et al. have found that both chondrogenic and osteogenic differentiation of adipose-derived adult mesenchymal stem cells are inhibited when these mesenchymal cells are induced to undergo differentiation *in vitro* at 2% O_2_ (Malladi et al. 2006). Nonetheless, targeted deletion of *HIF-1α* in adipose-derived adultmesenchymal stem cells results in reduced chondrogenic growth *in vitro* and decreased expression of chondrogenic genes such as *Sox 9* and *collagen* II (Malladi et al. 2007). These discrepancies could potentially be appreciated from at least two perspectives. (1) Chondrogenic differentiation is differentially regulated by HIF-dependent and -independent pathways under hypoxia. It is also worth noting that HIF-1α can be regulated by hypoxia-independent mechanisms (Harris, 2002; Semenza, 2003). (2) Cell fate decisions are likely determined as a result of interactions between hypoxia and other extracelluar stresses *in vivo*, especially during embryonic development. Additional study will be needed to fully understand the interactions between hypoxia-activated pathways and other stress-induced pathways that regulate differentiation and maintenance of stem cells.

## Concluding Remarks

Aerobic life on earth is thought to have begun some 2 billion years ago when the primitive atmosphere was hypoxic with approximately 2%–3% O_2_ (Han and Runnegar, 1992; Massabuau, 2003). A rapid rise in atmospheric O_2_ is estimated to have occurred around 1 billion years ago and to reach the present day level of 21% for the first time around 540 million years ago (Massabuau, 2003). The last 500 million years have witnessed a dramatic fluctuation in atmospheric O_2_ between 35% and 15% (Berner et al. 2007; Massabuau, 2003). The rise and fall of atmospheric O_2_ is closely tied to biological evolution on earth from speciation to extinction (Berner et al. 2007). Over the past 205 million years, atmospheric O_2_ rose from approximately 11% to the current level of 21%. The latest rise in atmospheric O_2_ is thought to be a key factor during the evolution of large placental mammals in the Cenozoic Era (Falkowski et al. 2005). Although rising O_2_ tension has been linked to increases in animal body size (Berner et al. 2007), it is quite remarkable that O_2_ tensions at the cellular level remain in a low range, independent of inspired O_2_ tensions (Massabuau, 2003). The role of O_2_ in evolution is yet to be determined, which further underscores the importance of O_2_ biology. Investigation of O_2_-dependent signal transduction will lead to the discovery of new cellular mechanisms that govern cellular growth, differentiation, and senescence, which will in turn provide insight into cellular mechanisms of evolution.
